# Integration of Synaptic Vesicle Cargo Retrieval with Endocytosis at Central Nerve Terminals

**DOI:** 10.3389/fncel.2017.00234

**Published:** 2017-08-03

**Authors:** Michael A. Cousin

**Affiliations:** Centre for Integrative Physiology, University of Edinburgh Edinburgh, United Kingdom

**Keywords:** endocytosis, vesicle, cargo, clathrin, presynapse

## Abstract

Central nerve terminals contain a limited number of synaptic vesicles (SVs) which mediate the essential process of neurotransmitter release during their activity-dependent fusion. The rapid and accurate formation of new SVs with the appropriate cargo is essential to maintain neurotransmission in mammalian brain. Generating SVs containing the correct SV cargo with the appropriate stoichiometry is a significant challenge, especially when multiple modes of endocytosis exist in central nerve terminals, which occur at different locations within the nerve terminals. These endocytosis modes include ultrafast endocytosis, clathrin-mediated endocytosis (CME) and activity-dependent bulk endocytosis (ADBE) which are triggered by specific patterns of neuronal activity. This review article will assess the evidence for the role of classical adaptor protein complexes in SV retrieval, discuss the role of monomeric adaptors and how interactions between specific SV cargoes can facilitate retrieval. In addition it will consider the evidence for preassembled plasma membrane cargo complexes and their role in facilitating these endocytosis modes. Finally it will present a unifying model for cargo retrieval at the presynapse, which integrates endocytosis modes in time and space.

## Introduction

The basis of neuronal communication in mammalian brain is the activity-dependent release of chemical neurotransmitter from the presynapse (nerve terminal). This event is triggered by action potential-driven calcium influx, resulting in the fusion of neurotransmitter-containing synaptic vesicles (SVs). The pool of SVs that are available for fusion at any one time is highly limited (between 200–400) inside a typical small central nerve terminal (Rosenmund and Stevens, 1996; Wilhelm et al., 2014). Neurotransmission is sustained by the rapid and accurate reformation of these SVs via a series of endocytosis modes, some of which form SVs directly at the plasma membrane, and others via an endosomal intermediate. The reformation of SVs is a complex process, since in addition to generating an organelle with the correct size and shape, this organelle must also contain the correct complement of SV cargo with the correct stoichiometry. SV cargo retrieval from the presynaptic plasma membrane is particularly challenging, when one considers that a typical SV contains greater than 80 different proteins (Takamori et al., 2006) with copy numbers ranging from 1 to 70 molecules (Takamori et al., 2006; Wilhelm et al., 2014). Because of these strict requirements, central nerve terminals have evolved a series of cargo recognition/retrieval strategies that have been integrated into a range of discrete endocytosis modes. This review article will summarize these strategies and then discuss how some or all integrate with different modes of endocytosis.

## SV Cargo Clustering Mechanisms

### AP-2

The first step in SV cargo retrieval is their clustering at the plasma membrane. The best characterized mechanism for SV cargo selection and clustering is via the clathrin adaptor protein complex, adaptor protein complex-2 (AP-2). AP-2 is a cytoplasmic heterotetrameric complex formed from four separate polypeptides named adaptins (α, β, σ and μ; Paczkowski et al., 2015). AP-2 is thought to adopt a “closed” conformation in the cytosol with SV cargo recognition motifs concealed by an intramolecular interaction of μ2 adaptin (LΦD/EΦD/E, Φ refers to a bulky hydrophobic amino acid) with a clathrin recognition motif on β2 (Collins et al., 2002). This auto-inhibition is relieved on AP-2 binding to phosphatidylinositol (4,5) bisphosphate (PI(4,5)P_2_) at the plasma membrane and stabilized by both cargo binding (Jackson et al., 2010; Kelly et al., 2014) and other pioneer molecules such as eps15, FcHO2 and SGIP (Henne et al., 2010; Hollopeter et al., 2014; Ma et al., 2016). This interaction also releases clathrin cargo recognition motifs (LLNLD) allowing recruitment of clathrin to the cargo cluster (Kelly et al., 2014). A series of canonical motifs have been identified on SV cargo that are recognized by both the μ2 and β2 adaptin subunits of this complex (YxxΦ, D/ExxxLL, x refers to any amino acid, Kelly and Owen, 2011; Rao et al., 2012). These canonical motifs were first identified when examining clathrin-mediated endocytosis (CME) in non-neuronal cells, however almost all SV cargo (but not all) contains at least one of these motifs.

Robust evidence exists to support the importance of AP-2 in SV cargo clustering and subsequent retrieval. For example, disruption of AP-2 binding motifs on a variety of SV cargo result in either their stranding on the plasma membrane or their retarded retrieval during action potential stimulation (Grass et al., 2004; Voglmaier et al., 2006; Yao et al., 2010; Foss et al., 2013). Furthermore, redirection of AP-2 to mitochondria or its depletion (using either shRNA or conditional knockout) slows the retrieval of a variety of SV cargo during endocytosis (Kim and Ryan, 2009; Willox and Royle, 2012; Kononenko et al., 2014). The fact that SV cargo retrieval is slowed, but not arrested, suggests that AP-2-mediated clustering is not obligatory for this event however. Therefore, AP-2 appears to be a key component of the SV cargo clustering machinery and is also important for their efficient retrieval during endocytosis.

### Monomeric Adaptors

The majority of SV cargo contains canonical interaction motifs for AP-2 that facilitate their clustering at the plasma membrane. However, some do not, including the essential soluble N-ethylmaleimide sensitive factor attachment protein receptor (SNARE) protein synaptobrevin II (sybII). This suggests that other clustering and retrieval mechanisms must occur at nerve terminals to ensure accurate SV cargo retrieval. The helical SNARE motif of sybII is essential for SV fusion when complexed with the plasma membrane proteins syntaxin and SNAP-25 (Weber et al., 1998). However, in addition to this key role, the SNARE motifs of multiple synaptobrevin isoforms form a binding platform for the adaptor molecule CALM which controls their trafficking in non-neuronal cells (Miller et al., 2011). This led to the discovery that the SNARE motif of sybII is a recognition motif for not just CALM, but also the neuronal specific isoform AP180 (Koo et al., 2011). Depletion of AP180 by shRNA or abrogation of AP180 binding via mutagenesis of the SNARE motif both led to increased plasma membrane stranding of sybII in cultured neurons (Koo et al., 2011). The role for AP180 in sybII clustering and retrieval appears to be specific for this essential SV cargo, since sybII surface stranding and retrieval was specifically disrupted in AP180 knockout mice, whereas other SV cargoes were unaffected (Koo et al., 2015). Thus AP180 and CALM are monomeric adaptor proteins that specially control sybII retrieval in central nerve terminals.

The existence of a monomeric adaptor for one specific SV cargo is not confined to sybII. A similar adaptation is also present in central nerve terminals for the essential SV cargo synaptotagmin-1 (Syt-1). Syt-1 is essential for coupling of calcium influx to SV fusion, with calcium binding to its C2 domains resulting in synchronous neurotransmitter release on action potential stimulation (Geppert et al., 1994). Syt-1 also contains a well characterized interaction motif for AP-2 within its C2B calcium binding domain (Zhang et al., 1994; Chapman et al., 1998; Grass et al., 2004). However, it also interacts with the monomeric adaptor protein stonin-2 via both its C2A and C2B domains (Walther et al., 2004; Diril et al., 2006). Ablation of multiple stonin-2 interaction interfaces on Syt-1 results in its increased surface stranding, suggesting this interaction is important for efficient clustering for its retrieval (Diril et al., 2006). Furthermore, increased Syt-1 stranding is observed in neurones derived from stonin-2 knockout mice with this effect specific for Syt-1, similar to the relationship between sybII and AP180 (Kononenko et al., 2013). Thus even though some SV cargo have AP-2 interaction motifs, potentially key SV molecules such as Syt-1 have additional levels redundancy for their clustering to ensure their accurate retrieval.

### Intrinsic Trafficking Partners (iTRAPs)

As discussed above, additional mechanisms exist in central nerve terminals to ensure that SV cargoes which perform essential functions in SV exocytosis, such as sybII and Syt-1, are clustered and retrieved efficiently during endocytosis. It is now becoming apparent that in addition to monomeric adaptors, interactions between specific SV cargoes themselves provide a final “fail-safe” mechanism to ensure accurate clustering and retrieval. The SV cargoes that provide this additional level of redundancy have been termed intrinsic trafficking partners (iTRAPs).

The first iTRAP identified was the abundant transmembrane SV cargo synaptophysin. Early studies which examined the effect of genomic deletion of the synaptophysin gene revealed that its absence had no apparent effect on either neurotransmitter release or presynaptic morphology (Eshkind and Leube, 1995; McMahon et al., 1996). Later studies revealed that the absence of synaptophysin had a small effect on endocytosis kinetics (Kwon and Chapman, 2011). However, the major presynaptic role of synaptophysin is the retention and clustering of sybII at the nerve terminal to ensure its accurate retrieval. In synaptophysin knockout neurons, both endogenous and exogenously expressed sybII is mislocalized from nerve terminals and is stranded at the plasma membrane (Gordon et al., 2011; Gordon and Cousin, 2013). Synaptophysin may also be essential for ensuring the correct stoichiometry of sybII trafficking. This was revealed via the titration of additional synaptophysin copies into wild-type neurons resulting in the clustering and retrieval of sybII in an 1:2 ratio (Gordon et al., 2016).

A second iTRAP is synaptic vesicle protein 2A (SV2A), which ensures the accurate clustering and retrieval of Syt-1. SV2A interacts with the C2B domain of Syt-1 via its N-terminus (Schivell et al., 1996) however this interaction only occurs when the N-terminus is phosphorylated at a specific residue (Thr 84) by the casein kinase family (Pyle et al., 2000; Zhang et al., 2015). Interestingly, the interaction motif for SV2A binding within the Syt-1 C2B domain overlaps with the interaction site for AP-2 (Zhang et al., 2015), suggesting that this interaction may be mutually exclusive. Depletion of SV2A with either shRNA or via genomic knockout results in an increased surface localization of Syt-1, with no discernible effect on other SV cargo (Yao et al., 2010; Zhang et al., 2015). Interestingly, SV2A depletion with shRNA in stonin-2 knockout neurons results in additive effects on Syt-1 plasma membrane stranding, suggesting both act in concert to control its clustering after exocytosis (Kaempf et al., 2015).

The iTRAPs synaptophysin and SV2A share a number of common features. First, they are both multi-pass transmembrane proteins, whereas the SV cargoes that they interact with are single-pass transmembrane proteins. Second, they are both present in an approximately 1:2 ratio with their interaction partners (Takamori et al., 2006; Wilhelm et al., 2014), suggesting that they may be the major determinants of cargo stoichiometry on SVs. Finally, they appear to function in concert with a monomeric adaptor protein. Is it still to be determined whether more iTRAPs exist, or whether they are limited to the trafficking of essential SV cargo sybII and Syt-1.

## Endocytosis Modes at The Presynapse

There are at least three different modes of endocytosis that form SVs, CME, ultrafast endocytosis and activity-dependent bulk endocytosis (ADBE), with the endocytosis mode utilized determined by the intensity of stimulation encountered.

### CME

The adaptor protein complex AP-2 clusters SV cargo on the plasma membrane and this action aids the recruitment of clathrin (Paczkowski et al., 2015). Since AP-2 is a major SV cargo clustering molecule, it suggests that the ubiquitous endocytosis mode CME should play a key role in SV reformation during neuronal activity. This assumption was supported by a number of key studies demonstrating that depletion of clathrin heavy chain, redirection of clathrin to mitochondria or overexpression of dominant negative clathrin adaptors arrested the retrieval of genetically-encoded reporters of SV cargo (Granseth et al., 2006; Willox and Royle, 2012). The requirement for CME appeared to be dependent on stimulus intensity, with CME being the dominant endocytosis mode during mild stimulation but saturating during more intense stimulation (Granseth et al., 2006; Clayton et al., 2008). An obligatory requirement for clathrin during endocytosis from the plasma membrane has recently been questioned (Kononenko et al., 2014; Watanabe et al., 2014; Soykan et al., 2017). However it is most likely that its role is more nuanced, with other studies revealing a contribution to both SV and cargo retrieval in different neuronal systems at physiological temperatures (Nicholson-Fish et al., 2015; Delvendahl et al., 2016).

### Ultrafast Endocytosis

As stated above, CME forms SVs directly from the plasma membrane. In contrast, ultrafast endocytosis forms small endosomes (approximately 60 nm in diameter) directly from the nerve terminal peri-active zone within 100 ms of action potential stimulation (Watanabe and Boucrot, 2017). SVs are then rapidly generated from these endosomes (within 5 s). Similar to CME, ultrafast endocytosis may saturate during periods of elevated neuronal activity (Soykan et al., 2017). Ultrafast endocytosis was discovered using a “flash-and-freeze” technique which integrated optogenetic stimulation with rapid freezing of tissue to resolve endocytic intermediates (Watanabe et al., 2013a,b). These studies were all performed at physiological temperature and under these conditions no clathrin intermediates were observed at the plasma membrane after stimulation (Watanabe et al., 2014). This suggested that ultrafast endocytosis rather than CME was the dominant endocytosis mode during mild stimulation. A lack of requirement for clathrin in ultrafast endocytosis was confirmed in neurons which had clathrin heavy chain depleted using siRNA (Watanabe et al., 2014). However, an obligatory requirement for clathrin in SV generation from these endosomes was revealed under the same experimental conditions (Watanabe et al., 2014).

### ADBE

The final presynaptic endocytosis mode is ADBE. As the name suggests, ADBE is only triggered during high activity and is the dominant endocytosis mode under these conditions (Clayton et al., 2008). Similar to ultrafast endocytosis, ADBE is a two-step process with bulk endosomes being formed direct from the plasma membrane, from which SVs are subsequently generated (Clayton and Cousin, 2009). In contrast to ultrafast endocytosis however, ADBE forms large endosomes (up to 500 μm diameter) with a timescale that is at least an order of magnitude slower (Clayton et al., 2008). The formation of bulk endocytosis is directly coupled to neuronal activity via a dephosphorylation cascade initiated by the protein phosphatase calcineurin (Clayton et al., 2009; Wu et al., 2014). Bulk endosome formation is also clathrin-independent (Heerssen et al., 2008; Kasprowicz et al., 2008; Kononenko et al., 2014; Nicholson-Fish et al., 2015; Soykan et al., 2017), however SV generation from these endosomes requires both clathrin and adaptor proteins (Heerssen et al., 2008; Kasprowicz et al., 2008; Cheung and Cousin, 2012; Kononenko et al., 2014).

## How Does Cargo Clustering Integrate with Known Endocytosis Modes?

Multiple SV cargo clustering mechanisms exist in central nerve terminals. In addition to this there are at least three discrete endocytosis modes that can be triggered dependent on stimulus intensity. This raises an important question—do different endocytosis modes have similar or autonomous mechanisms that are responsible for cargo selection and retrieval?

### Pre-Assembled Clusters—Separating Fused SVs from Cargo Retrieval

Ultrafast endocytosis retrieves plasma membrane at the peri-active zone within 50 ms of action potential stimulation (Watanabe et al., 2013b). Therefore, it is highly unlikely that the same cargoes that were present on the SV that fused during the same stimulation train are retrieved via this endocytosis mode. This is a major conceptual point, since it illustrates that even when exocytosis and endocytosis are in balance, the SV cargo deposited and retrieved from the plasma membrane can be distinct. In support, there is now accumulating experimental evidence that plasma membrane cargo is clustered prior to action potential stimulation and can be distinct from that deposited during exocytosis.

The first evidence for the absence of a mandatory requirement for newly deposited SV cargo to be retrieved by endocytosis came from studies using a genetically encoded reporter of SV cargo that had a protease-cleavable site (sybII-pHluorin). Removal of the fluorescent moiety from the plasma membrane population of sybII-pHluorin revealed that the reporter deposited on the cell surface during neuronal activity was not the same as the reporter that was retrieved by endocytosis (Wienisch and Klingauf, 2006). This result was confirmed in a separate study which demonstrated that endogenous SV cargoes present on the plasma membrane were retrieved during endocytosis (Fernández-Alfonso et al., 2006). Furthermore, plasma membrane-localized SV cargoes were demonstrated to be preferentially retrieved during the same stimulus train though use of antibodies conjugated to fluorescent pH-sensitive dyes that tracked endogenous proteins. Such pre-assembled plasma membrane clusters have been termed the “readily retrievable pool” and were revealed to reside in the peri-active zone (Hua et al., 2011). It is therefore tempting to speculate that this “readily retrievable pool” forms the cargo that is available and immediately retrieved during ultrafast endocytosis.

SV cargo should be freely diffusible in the plasma membrane, meaning the readily retrievable pool has to be constrained within the peri-active zone area via specific molecular mechanisms. Adaptor proteins may perform a key role in this regard. Using genetically-encoded reporters of SV cargo in conjunction with super-resolution microscopy, it was demonstrated that freshly deposited cargo was freely diffusible within the plasma membrane, but was prevented from leaving the presynapse by the presence of adaptor proteins and potentially other unidentified factors which retarded diffusion (Gimber et al., 2015). Interestingly, the iTRAP synaptophysin has recently been proposed to aid clearance of sybII from active zone regions during action potential stimulation (Rajappa et al., 2016). This suggests that a number of SV cargo clustering mechanisms participate in forming and maintaining the readily retrievable pool.

### Ultrafast Endocytosis vs. Kiss-and-Run

The premise that different SV cargoes are retrieved during ultrafast endocytosis that were deposited in the plasma membrane has invited comparisons with a different endocytosis mode that is widely utilized in secretory cells—kiss-and-run (Alés et al., 1999; Wen et al., 2016). During kiss-and-run the secretory vesicle transiently fuses to release its soluble contents and then immediately fissions, without ever integrating into the plasma membrane (Alabi and Tsien, 2013). The existence of kiss-and-run at typical small central nerve terminals is still contested (He and Wu, 2007), mainly due to the fact that very few methods exist to visualize the occurrence of this potential event. The major argument for kiss-and-run in neurons was conceptual, in that it appeared to provide a mechanism to regenerate SVs with high speed and minimal energy expenditure. However the discovery of ultrafast endocytosis may negate a number of these conceptual points.

For example, ultrafast endocytosis can generate SVs within 3–5 s of the invasion of action potentials. While still an order of magnitude slower that kiss-and-run (Alés et al., 1999), it should be considered that the kinetics of SV acidification and subsequent neurotransmitter filling would be rate limiting in terms of producing a fully functional SV (Hori and Takahashi, 2012). This specific point may have a large impact on presynaptic physiology, since SVs generated via ultrafast endocytosis may by fully fusion competent, but incompletely filled with neurotransmitter. Interestingly a recent study has suggested that incompletely filled SVs are less fusion competent than fully filled SVs (Rost et al., 2015), suggesting that ultrafast SVs may not immediately replenish the RRP, or if they do so, will decrease release probability. It will therefore be critical to determine the fusion competence of SVs generated via ultrafast endocytosis and whether this competence increases over time.

The discovery of ultrafast endocytosis also removes another conceptual argument for kiss-and-run, the requirement to keep the active zone clear of deposited SV cargo. Theoretically, kiss-and-run should facilitate SV fusion in this regard, since no cargo physically enters the active zone. However, a number of studies have demonstrated that interfering with the function of dynamin, clathrin or the iTRAP synaptophysin results in a short-term depression of release, suggesting SV cargo was being deposited into the active zone (Kawasaki et al., 2000; Hua et al., 2013; Rajappa et al., 2016). By immediately removing the preassembled readily retrievable pool of clustered SV cargo, ultrafast endocytosis should permit the fast clearance of cargo by facilitating new clustering within the now empty periactive zone area.

Since ultrafast endocytosis fulfils most of the conceptual requirements that were proposed or the existence of kiss-and-run, it is highly plausible that the latter may not be utilized in small central nerve terminals. Increasing knowledge of the molecular mechanism of ultrafast endocytosis should allow this hypothesis to be tested directly, via molecular intervention in large secretory cells where kiss-and-run is prevalent. The converse is also true, if future studies in these cells identify key molecules for kiss-and-run, the function of which can then be interrogated at central synapses.

### Clathrin-Independent or -Dependent Retrieval? Differences in Space and Time

Clathrin-dependent mechanisms are required for the generation of SVs in all three presynaptic endocytosis modes regardless of whether they occur at the plasma membrane or endosome level. Therefore, while some modes are referred to as “clathrin-independent” this is something of a misnomer, since clathrin is essential in all cases at some stage. The observed differential locational requirement for clathrin may not even be a question of mechanism, but simply a result of speed—specifically the time required to form a clathrin coat. In ultrafast endocytosis for example, endosomes are formed too rapidly to allow a clathrin coat to generate SVs from the readily retrievable pool. This may also be the case for ADBE, since large invaginations rapidly occur and then fission at the very onset of activity (Wu and Wu, 2007; Clayton et al., 2008). Alternatively, the clathrin-independence of bulk endosome formation during ADBE may arise from the fact that this mode is most likely to occur distal to the peri-active zone and therefore away from preassembled AP-2 adaptor complexes. Therefore, the clathrin-independence of both ultrafast endocytosis and ADBE may be due to either the speed or location of endosome formation. The reduced contribution of clathrin-dependent mechanisms to SV cargo retrieval from the plasma membrane at physiological temperatures (Kononenko et al., 2014; Watanabe et al., 2014; Nicholson-Fish et al., 2015; Delvendahl et al., 2016; Soykan et al., 2017) is an extension of this reasoning, since SV generation via clathrin will be rate limiting, when compared to ultrafast and ADBE functioning at their optimal kinetics.

As stated above, clathrin is essential to generate SVs in all forms of presynaptic endocytosis. Clathrin is recruited to adaptor protein complexes during the binding of SV cargo (Kelly et al., 2014), suggesting adaptor protein complexes such as AP-2 also play a pivotal role. Inducible knockout of AP-2 both in neuronal culture and *in vivo* resulted in enlarged bulk endosomes and SV depletion in nerve terminals (Kononenko et al., 2014), suggesting AP-2 is required for SV generation during high intensity stimulation. The endosomal adaptor protein complexes AP-1 and AP-3 are also required for SV generation from bulk endosomes, since either interference with their recruitment or their depletion with shRNA arrested SV generation (Cheung and Cousin, 2012). This diversity of adaptor protein complexes may reflect the heterogeneity in lipid composition of the bulk endosome. In this regard, AP-2 will continue to cluster SV cargo recently internalized from the plasma membrane (which is high in PI(4,5)P_2_), whereas the ongoing destruction of this phospholipid to make the lipid composition more “endosomal” (Chang-Ileto et al., 2011; Milosevic et al., 2011) may require AP-1 and AP-3 to maintain SV cargo clustering.

Different adaptor protein complexes have different selectivity for specific SV cargo (Robinson, 2004; Newell-Litwa et al., 2007). Therefore, the fact that a combination of adaptor protein complexes are required for SV generation at bulk endosomes suggests SVs that are formed by ADBE may have a distinct molecular composition in terms their cargo to those formed via CME (which will be exclusively AP-2-dependent). In support, SVs generated via ADBE replenish specific SV pools and perform specific roles within central nerve terminals, such as replenishment of the reserve SV pool and asynchronous release (Cheung et al., 2010; Raingo et al., 2012; Evstratova et al., 2014; Nicholson-Fish et al., 2015; Li et al., 2017).

### Cargo Retrieval during ADBE

The process of ADBE involves a large internalization of plasma membrane, presumably a distance away from the active zone. Therefore, would SV cargo that is clustered at the peri-active zone be internalized on formation of bulk endosomes, and if not how would essential cargo be incorporated into SVs generated via ADBE? SVs that are generated by ADBE are fusion competent (Cheung et al., 2010; Cheung and Cousin, 2012, 2013; Evstratova et al., 2014), meaning essential cargo has been integrated into SVs at some stage of the process. There are two extreme possibilities. First, SV cargo destined for ADBE is clustered for internalization at sites distant from the peri-active zone, or second, plasma membrane molecules (including SV cargo) are retrieved in a non-specific manner during the rapid invagination that occurs during bulk endosome formation.

Biochemical enrichment of bulk endosomes immediately after their formation revealed that a series of essential SV cargo molecules were present, suggesting they were retrieved during invagination (Nicholson-Fish et al., 2015). Furthermore, simultaneous monitoring of endogenous SV cargo with membrane invagination in large atypical nerve terminals demonstrated that during “fast” endocytosis cargo was internalized into a slowly acidifying compartment (Okamoto et al., 2016). Fast endocytosis is triggered by strong stimulation and generally thought to equate to ADBE (Wu et al., 2005), therefore this compartment may reflect bulk endosomes. In contrast, when the retrieval of four different exogenously expressed reporters of SV cargo (sybII, Syt-1, vGLUT and synaptophysin) were examined during high intensity activity in typical small nerve terminals, there was no evidence of sequestration into a slowly acidifying compartment (Nicholson-Fish et al., 2015). Furthermore, the retrieval of these cargoes was unaffected by any maneuver that arrested ADBE (Nicholson-Fish et al., 2015). The presence of endogenous SV cargo on purified bulk endosomes and their efficient retrieval during intense activity suggests that these essential molecules are sequestered via ADBE. However, it also suggests that exogenous reporters of SV cargo may not be accurately reflecting the physiological situation (Nicholson-Fish et al., 2015; Okamoto et al., 2016).

In contrast to other exogenously expressed reporters, VAMP4-pHluorin was retrieved by ADBE. VAMP4 is a non-canonical SNARE that displays a relatively high plasma membrane localization (Raingo et al., 2012). Endogenous VAMP4 was highly enriched on purified bulk endosomes and its retrieval during high intensity stimulation was arrested by interventions that inhibited ADBE but not CME (Nicholson-Fish et al., 2015). Interestingly, depletion of VAMP4 itself arrested ADBE, indicating that it is an essential molecule for bulk endosome generation. The underlying mechanism has not yet been determined, however VAMP4 lacking a dileucine motif for AP-1 (Peden et al., 2001) could not support ADBE when it replaced the endogenous form in primary cultures (Nicholson-Fish et al., 2015). Thus, VAMP4 may act as a nucleating factor for ADBE cargo in locations distinct from the peri-active zone. Interestingly, synaptotagmin-7 (Syt-7) displays a very similar trafficking behavior to VAMP4 (Li et al., 2017), suggesting it may also be selectively retrieved during ADBE. In support, both VAMP4 and Syt-7 perform key roles in asynchronous release (Raingo et al., 2012; Bacaj et al., 2013), suggesting ADBE derived SVs are essential to mediate this form of neurotransmitter release (Nicholson-Fish et al., 2015; Li et al., 2017).

## Perspectives

### Does the Amount of Cargo on the Plasma Membrane Equate with Retrieval?

Some proportion of SV cargo will always reside on the plasma membrane at any one time, however it is striking that there are large discrepancies between the amounts observed in resting neurons. Some SV cargoes display extremely low plasma membrane expression (such as vGLUT, vGAT and SV2A) whereas others (such as Syt-1, sybII, Syt-7, Vti1a and VAMP4) display expression levels of up to 50% of total (Raingo et al., 2012; Ramirez et al., 2012; Santos et al., 2013; Pan et al., 2015; Li et al., 2017). A key question is—why is there such a disparity between these cargoes? One possible explanation is that cargoes with a low SV copy number are more efficiently clustered than those with a high copy number such as sybII and Syt-1. However, there is no obvious linear relationship between plasma membrane expression and this parameter (Takamori et al., 2006; Wilhelm et al., 2014; Pan et al., 2015). Alternatively, the high plasma membrane expression of sybII and Syt-1 may also reflect the fact that these proteins are essential for SV fusion. Their high preponderance at the plasma membrane will therefore provide a reservoir from which endocytosis can sample to sustain efficient neurotransmission during periods of high activity (Fernández-Alfonso et al., 2006). There may also be mechanistic requirements for high plasma membrane expression of specific cargoes, for example the essential requirement for VAMP4 in ADBE (Nicholson-Fish et al., 2015). Experimental strategies to anchor the cytoplasmic domains of SV cargo in the plasma membrane are available (Yao et al., 2012) and could be employed in future to determine whether the permanent residency of SV cargo has any mechanistic impact on either clustering or endocytosis when expressed in wild-type systems.

An increase in the amount of plasma membrane localized SV cargoes is usually assumed to reflect a deficiency in their retrieval during endocytosis. Many studies are consistent with this hypothesis (Kim and Ryan, 2009; Gordon et al., 2011; Foss et al., 2013; Koo et al., 2015). However in specific cases, there seems to be a disconnection between plasma membrane localization and SV cargo retrieval. The most obvious instance is the activity-dependent retrieval of Syt-1. As stated previously, depletion of either AP-2, stonin-2 or SV2A all result in the increased stranding of Syt-1 at the plasma membrane (Kim and Ryan, 2009; Yao et al., 2010; Kononenko et al., 2013; Kaempf et al., 2015; Zhang et al., 2015). In agreement with the studies above, depletion of AP-2 using shRNA also slows the retrieval of Syt-1 in mammalian neurones (Kim and Ryan, 2009). However, genomic knockout of stonin-2, or depletion of SV2A with shRNA accelerates the retrieval of Syt-1 (Kononenko et al., 2013; Zhang et al., 2015) with this effect exacerbated when SV2A is depleted in stonin-2 knockout neurones (Kaempf et al., 2015). There are a number of potential explanations for this disengagement between surface localization and retrieval. First there may be different retrieval mechanisms that are dominant at rest or during evoked neuronal activity. Thus, depletion of stonin-2 or SV2A may impact one particular endocytosis mode resulting in accelerated Syt-1 retrieval. In support, stonin-2 knockout neurons display in decrease in large endosomes, suggesting a defect in ADBE (Kononenko et al., 2013). However, depletion of SV2A has no discernible effect on endocytosis (Yao et al., 2010; Zhang et al., 2015). Alternatively, faulty Syt-1 clustering due to the absence of stonin-2 or SV2A may provide the illusion of accelerated Syt-1 retrieval. In this scenario SV cargo which escapes the peri-active zone is not available for retrieval. The remaining Syt-1 (which is inefficiently clustered by AP-2) will therefore appear to be more efficiently retrieved. This dichotomy at present is restricted to Syt-1, since depletion of AP-2, AP180 or synaptophysin all increase sybII surface expression and retard its retrieval (Kim and Ryan, 2009; Gordon et al., 2011; Koo et al., 2011, 2015). Therefore, this phenotype is not specific to cargoes that are trafficked by monomeric adaptors or iTRAPs.

### SV Cargo Retrieval as an Entry Mechanism

The physiological role of SV cargo retrieval via endocytosis is to sustain neurotransmission, however it can also be exploited by exogenous agents to gain access into neurons. An obvious example of this is the entry of clostridial neurotoxins such as tetanus and botulinum toxins. A number of SV cargoes have been proposed to bind to and mediate the entry of a series of toxin molecules (SV2 for tetanus toxin and botulinum toxins A, D, E, F; Syt-1 for botulinum toxins B, G; Dong et al., 2003, 2006, 2008; Fu et al., 2009; Rummel et al., 2009; Yeh et al., 2010; Peng et al., 2011). In addition to toxic effects, the luminal domains of SV cargo can also act as facilitators of drug entry into neurons. A classic example is the widely employed anti-epileptic levetiracetam (Lynch et al., 2004). Levetiracetam binds SV2A at a series of transmembrane domains (Correa-Basurto et al., 2015; Lee et al., 2015) and its uptake is facilitated by increased neuronal activity (Meehan et al., 2011, 2012). Therefore SV cargo retrieval may offer an interesting clinical avenue, by linking the therapeutic payload to small molecules which recognize the luminal domains of SV cargoes.

### Integration of Efficient SV Cargo Retrieval and Endocytosis

The efficient incorporation of the correct cargo with the appropriate stoichiometry into SVs is essential for optimal presynaptic performance. Current understanding of cargo clustering mechanisms at the plasma membrane are continually evolving and are being integrated into recently identified mechanisms of endocytosis, some of which are only beginning to be understood. This presents a challenge when attempting to understand how SV cargo is selected and how SVs are generated from the plasma membrane. The model presented below attempts to incorporate current information to present a unified framework for the retrieval of both cargo and SVs during neuronal activity.

The presynapse has always been thought of as a primed system, with SVs that are competent for fusion being docked at the active zone. The trigger for neurotransmitter release is invasion of an action potential, resulting in calcium influx and SV fusion. This priming analogy can now be extrapolated to endocytosis, with preassembled clusters of SV cargo primed for retrieval in the readily retrievable pool. Therefore almost immediately on action potential invasion this cargo is internalized by ultrafast endocytosis, providing a rapid mechanism via which SVs with the correct complement of cargo can be generated.

**Figure 1:**
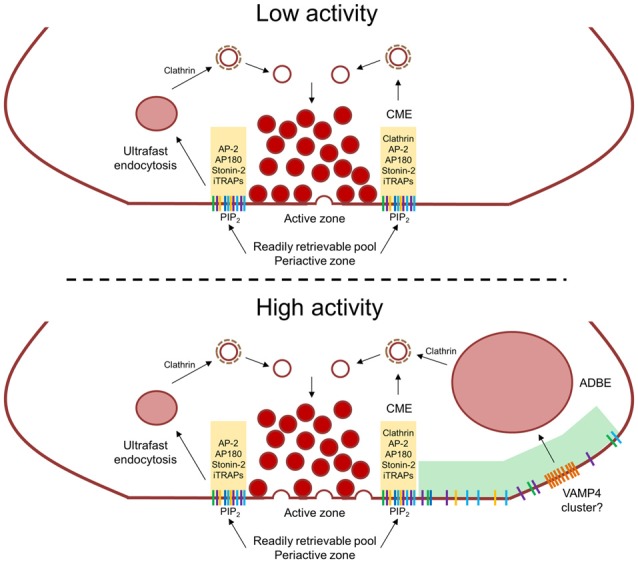
Activity-dependent control of synaptic vesicle (SV) cargo clustering and retrieval. During low neuronal activity SV cargo is clustered within the peri-active zone by AP-2, the monomeric adaptors AP180 and stonin-2 and intrinsic trafficking partners (iTRAPs), This constitutes the “readily retrievable pool” (yellow shaded area) and is immediately retrieved by either ultrafast endocytosis or clathrin-mediated endocytosis (CME). Due to their different kinetics, clathrin forms SVs from endosomes during ultrafast endocytosis whereas it forms SVs at the plasma membrane during CME. During high neuronal activity, an increased number of fusion events saturates the readily retrievable pool, meaning SV cargo escapes the peri-active zone. This cargo is then captured by activity-dependent bulk endocytosis (ADBE) which forms endosomes directly from the plasma membrane at sites distal to the active zone (green shaded are). There may also be ADBE-specific cargoes (such as VAMP4) that are clustered of within this region which will also be specifically internalized via ADBE.

The process of cargo reclustering at the peri-active should occur as soon as SVs fuse on action potential invasion. This is important for: (1) preparing the readily retrievable pool for the next cycle of activity (Gimber et al., 2015); and (2) for clearing the active zone for new fusion events (Hua et al., 2013; Rajappa et al., 2016). During low levels of activity this clustering will be sufficient to replenish SVs generated from either ultrafast endosomes or via CME at the plasma membrane (Figure 1). However, during more intense stimulation it is highly likely that the readily retrievable pool will be become saturated by the flux of SV cargo being deposited into the plasma membrane. In this instance, essential SV cargo will escape the confines of the peri-active zone and instead be accumulated by ADBE (which occurs distal to the peri-active zone and will only be triggered under intense stimulation conditions, Figure 1). This may explain why bulk endosomes accumulate endogenous SV cargo (Nicholson-Fish et al., 2015; Okamoto et al., 2016) and the requirement for additional adaptor protein complexes for their clustering at the bulk endosome membrane (Cheung and Cousin, 2012; Kononenko et al., 2014).

In addition to classical SV cargoes, non-canonical forms such as VAMP4 and Syt-7 may cluster (or be clustered by an as yet unidentified factor) ADBE-specific cargo at sites selective for ADBE (Figure 1). The location of such sites is still undetermined, however may link to sites of actin polymerization related to the action of both formins and myosin II (Gormal et al., 2015; Soykan et al., 2017). The essential role of VAMP4 interactions with adaptor proteins in generating bulk endosomes in ADBE (Nicholson-Fish et al., 2015) suggests that it may be a key molecule in nucleating this specific subset of cargo molecules. Whether ADBE has specific adaptor molecules to perform this task remains to be determined, possibly cargo—cargo interactions similar to those observed with iTRAPs may be sufficient in this instance.

## Author Contributions

MAC conceived and wrote the manuscript.

## Conflict of Interest Statement

The author declares that the research was conducted in the absence of any commercial or financial relationships that could be construed as a potential conflict of interest.

## References

[B1] Alabi A. A. Tsien R. W. (2013). Perspectives on kiss-and-run: role in exocytosis, endocytosis, and neurotransmission. Ann. Rev. Physiol. 75, 393–422. 10.1146/annurev-physiol-020911-153305 23245563

[B2] Alés E. Tabares L. Poyato J. M. Valero V. Lindau M. Alvarez de Toledo G. (1999). High calcium concentrations shift the mode of exocytosis to the kiss-and-run mechanism. Nat. Cell Biol. 1, 40–44. 10.1038/9012 10559862

[B3] Bacaj T. Wu D. Yang X. Morishita W. Zhou P. Xu W.. (2013). Synaptotagmin-1 and synaptotagmin-7 trigger synchronous and asynchronous phases of neurotransmitter release. Neuron 80, 947–959. 10.1016/j.neuron.2013.10.026 24267651 PMC3888870

[B4] Chang-Ileto B. Frere S. G. Chan R. B. Voronov S. V. Roux A. Di Paolo G. (2011). Synaptojanin 1-mediated PI(4,5)P2 hydrolysis is modulated by membrane curvature and facilitates membrane fission. Dev. Cell 20, 206–218. 10.1016/j.devcel.2010.12.008 21316588 PMC3058127

[B5] Chapman E. R. Desai R. C. Davis A. F. Tornehl C. K. (1998). Delineation of the oligomerization, AP-2 binding, and synprint binding region of the C2B domain of synaptotagmin. J. Biol. Chem. 273, 32966–32972. 10.1074/jbc.273.49.32966 9830048

[B6] Cheung G. Cousin M. A. (2012). Adaptor protein complexes 1 and 3 are essential for generation of synaptic vesicles from activity-dependent bulk endosomes. J. Neurosci. 32, 6014–6023. 10.1523/JNEUROSCI.6305-11.2012 22539861 PMC3348540

[B7] Cheung G. Cousin M. A. (2013). Synaptic vesicle generation from activity-dependent bulk endosomes requires calcium and calcineurin. J. Neurosci. 33, 3370–3379. 10.1523/JNEUROSCI.4697-12.2013 23426665 PMC3589713

[B8] Cheung G. Jupp O. J. Cousin M. A. (2010). Activity-dependent bulk endocytosis and clathrin-dependent endocytosis replenish specific synaptic vesicle pools in central nerve terminals. J. Neurosci. 30, 8151–8161. 10.1523/JNEUROSCI.0293-10.2010 20554865 PMC2889610

[B9] Clayton E. L. Anggono V. Smillie K. J. Chau N. Robinson P. J. Cousin M. A. (2009). The phospho-dependent dynamin-syndapin interaction triggers activity-dependent bulk endocytosis of synaptic vesicles. J. Neurosci. 29, 7706–7717. 10.1523/JNEUROSCI.1976-09.2009 19535582 PMC2713864

[B10] Clayton E. L. Cousin M. A. (2009). The molecular physiology of activity-dependent bulk endocytosis of synaptic vesicles. J. Neurochem. 111, 901–914. 10.1111/j.1471-4159.2009.06384.x 19765184 PMC2871311

[B11] Clayton E. L. Evans G. J. Cousin M. A. (2008). Bulk synaptic vesicle endocytosis is rapidly triggered during strong stimulation. J. Neurosci. 28, 6627–6632. 10.1523/JNEUROSCI.1445-08.2008 18579735 PMC2588494

[B12] Collins B. M. McCoy A. J. Kent H. M. Evans P. R. Owen D. J. (2002). Molecular architecture and functional model of the endocytic AP2 complex. Cell 109, 523–535. 10.1016/s0092-8674(02)00735-3 12086608

[B13] Correa-Basurto J. Cuevas-Hernández R. I. Phillips-Farfán B. V. Martínez-Archundia M. Romo-Mancillas A. Ramírez-Salinas G. L.. (2015). Identification of the antiepileptic racetam binding site in the synaptic vesicle protein 2A by molecular dynamics and docking simulations. Front. Cell. Neurosci. 9:125. 10.3389/fncel.2015.00125 25914622 PMC4392693

[B14] Delvendahl I. Vyleta N. P. von Gersdorff H. Hallermann S. (2016). Fast, temperature-sensitive and clathrin-independent endocytosis at central synapses. Neuron 90, 492–498. 10.1016/j.neuron.2016.03.013 27146271 PMC5125781

[B15] Diril M. K. Wienisch M. Jung N. Klingauf J. Haucke V. (2006). Stonin 2 is an AP-2-dependent endocytic sorting adaptor for synaptotagmin internalization and recycling. Dev. Cell 10, 233–244. 10.1016/j.devcel.2005.12.011 16459302

[B16] Dong M. Liu H. Tepp W. H. Johnson E. A. Janz R. Chapman E. R. (2008). Glycosylated SV2A and SV2B mediate the entry of botulinum neurotoxin E into neurons. Mol. Biol. Cell 19, 5226–5237. 10.1091/mbc.E08-07-0765 18815274 PMC2592654

[B17] Dong M. Richards D. A. Goodnough M. C. Tepp W. H. Johnson E. A. Chapman E. R. (2003). Synaptotagmins I and II mediate entry of botulinum neurotoxin B into cells. J. Cell Biol. 162, 1293–1303. 10.1083/jcb.200305098 14504267 PMC2173968

[B18] Dong M. Yeh F. Tepp W. H. Dean C. Johnson E. A. Janz R.. (2006). SV2 is the protein receptor for botulinum neurotoxin A. Science 312, 592–596. 10.1126/science.1123654 16543415

[B19] Eshkind L. G. Leube R. E. (1995). Mice lacking synaptophysin reproduce and form typical synaptic vesicles. Cell Tiss. Res. 282, 423–433. 10.1007/s004410050493 8581936

[B20] Evstratova A. Chamberland S. Faundez V. Tóth K. (2014). Vesicles derived via AP-3-dependent recycling contribute to asynchronous release and influence information transfer. Nat. Commun. 5:5530. 10.1038/ncomms6530 25410111 PMC4239664

[B21] Fernández-Alfonso T. Kwan R. Ryan T. A. (2006). Synaptic vesicles interchange their membrane proteins with a large surface reservoir during recycling. Neuron 51, 179–186. 10.1016/j.neuron.2006.06.008 16846853

[B22] Foss S. M. Li H. Santos M. S. Edwards R. H. Voglmaier S. M. (2013). Multiple dileucine-like motifs direct VGLUT1 trafficking. J. Neurosci. 33, 10647–10660. 10.1523/JNEUROSCI.5662-12.2013 23804088 PMC3693054

[B23] Fu Z. Chen C. Barbieri J. T. Kim J. J. Baldwin M. R. (2009). Glycosylated SV2 and gangliosides as dual receptors for botulinum neurotoxin serotype F. Biochemistry 48, 5631–5641. 10.1021/bi9002138 19476346 PMC2709598

[B24] Geppert M. Goda Y. Hammer R. E. Li C. Rosahl T. W. Stevens C. F.. (1994). Synaptotagmin I: a major Ca^2+^ sensor for transmitter release at a central synapse. Cell 79, 717–727. 10.1016/0092-8674(94)90556-8 7954835

[B25] Gimber N. Tadeus G. Maritzen T. Schmoranzer J. Haucke V. (2015). Diffusional spread and confinement of newly exocytosed synaptic vesicle proteins. Nat. Commun. 6:8392. 10.1038/ncomms9392 26399746 PMC4598626

[B26] Gordon S. L. Cousin M. A. (2013). X-linked intellectual disability-associated mutations in synaptophysin disrupt synaptobrevin II retrieval. J. Neurosci. 33, 13695–13700. 10.1523/JNEUROSCI.0636-13.2013 23966691 PMC3755716

[B27] Gordon S. L. Harper C. B. Smillie K. J. Cousin M. A. (2016). A fine balance of synaptophysin levels underlies efficient retrieval of synaptobrevin II to synaptic vesicles. PLoS One 11:e0149457. 10.1371/journal.pone.0149457 26871701 PMC4752265

[B28] Gordon S. L. Leube R. E. Cousin M. A. (2011). Synaptophysin is required for synaptobrevin retrieval during synaptic vesicle endocytosis. J. Neurosci. 31, 14032–14036. 10.1523/JNEUROSCI.3162-11.2011 21957264 PMC3188371

[B29] Gormal R. S. Nguyen T. H. Martin S. Papadopulos A. Meunier F. A. (2015). An acto-myosin II constricting ring initiates the fission of activity-dependent bulk endosomes in neurosecretory cells. J. Neurosci. 35, 1380–1389. 10.1523/JNEUROSCI.3228-14.2015 25632116 PMC6795266

[B30] Granseth B. Odermatt B. Royle S. J. Lagnado L. (2006). Clathrin-mediated endocytosis is the dominant mechanism of vesicle retrieval at hippocampal synapses. Neuron 51, 773–786. 10.1016/j.neuron.2006.08.029 16982422

[B31] Grass I. Thiel S. Höning S. Haucke V. (2004). Recognition of a basic AP-2 binding motif within the C2B domain of synaptotagmin is dependent on multimerization. J. Biol. Chem. 279, 54872–54880. 10.1074/jbc.M409995200 15491995

[B32] He L. Wu L. G. (2007). The debate on the kiss-and-run fusion at synapses. Trends Neurosci. 30, 447–455. 10.1016/j.tins.2007.06.012 17765328

[B33] Heerssen H. Fetter R. D. Davis G. W. (2008). Clathrin dependence of synaptic-vesicle formation at the *Drosophila* neuromuscular junction. Curr. Biol. 18, 401–409. 10.1016/j.cub.2008.02.055 18356056 PMC2699046

[B34] Henne W. M. Boucrot E. Meinecke M. Evergren E. Vallis Y. Mittal R.. (2010). FCHo proteins are nucleators of clathrin-mediated endocytosis. Science 328, 1281–1284. 10.1126/science.1188462 20448150 PMC2883440

[B35] Hollopeter G. Lange J. J. Zhang Y. Vu T. N. Gu M. Ailion M.. (2014). The membrane-associated proteins FCHo and SGIP are allosteric activators of the AP2 clathrin adaptor complex. Elife 3:03648. 10.7554/eLife.03648 25303366 PMC4215536

[B36] Hori T. Takahashi T. (2012). Kinetics of synaptic vesicle refilling with neurotransmitter glutamate. Neuron 76, 511–517. 10.1016/j.neuron.2012.08.013 23141063

[B37] Hua Y. Sinha R. Thiel C. S. Schmidt R. Huve J. Martens H.. (2011). A readily retrievable pool of synaptic vesicles. Nat. Neurosci. 14, 833–839. 10.1038/nn.2838 21666673

[B38] Hua Y. Woehler A. Kahms M. Haucke V. Neher E. Klingauf J. (2013). Blocking endocytosis enhances short-term synaptic depression under conditions of normal availability of vesicles. Neuron 80, 343–349. 10.1016/j.neuron.2013.08.010 24139039

[B39] Jackson L. P. Kelly B. T. McCoy A. J. Gaffry T. James L. C. Collins B. M.. (2010). A large-scale conformational change couples membrane recruitment to cargo binding in the AP2 clathrin adaptor complex. Cell 141, 1220–1229. 10.1016/j.cell.2010.05.006 20603002 PMC3655264

[B40] Kaempf N. Kochlamazashvili G. Puchkov D. Maritzen T. Bajjalieh S. M. Kononenko N. L.. (2015). Overlapping functions of stonin 2 and SV2 in sorting of the calcium sensor synaptotagmin 1 to synaptic vesicles. Proc. Natl. Acad. Sci. U S A 112, 7297–7302. 10.1073/pnas.1501627112 26015569 PMC4466747

[B41] Kasprowicz J. Kuenen S. Miskiewicz K. Habets R. L. Smitz L. Verstreken P. (2008). Inactivation of clathrin heavy chain inhibits synaptic recycling but allows bulk membrane uptake. J. Cell Biol. 182, 1007–1016. 10.1083/jcb.200804162 18762582 PMC2528586

[B42] Kawasaki F. Hazen M. Ordway R. W. (2000). Fast synaptic fatigue in shibire mutants reveals a rapid requirement for dynamin in synaptic vesicle membrane trafficking. Nat. Neurosci. 3, 859–860. 10.1038/78753 10966613

[B43] Kelly B. T. Graham S. C. Liska N. Dannhauser P. N. Höning S. Ungewickell E. J.. (2014). Clathrin adaptors. AP2 controls clathrin polymerization with a membrane-activated switch. Science 345, 459–463. 10.1126/science.1254836 25061211 PMC4333214

[B44] Kelly B. T. Owen D. J. (2011). Endocytic sorting of transmembrane protein cargo. Curr. Opin. Cell Biol. 23, 404–412. 10.1016/j.ceb.2011.03.004 21450449

[B45] Kim S. H. Ryan T. A. (2009). Synaptic vesicle recycling at CNS snapses without AP-2. J. Neurosci. 29, 3865–3874. 10.1523/JNEUROSCI.5639-08.2009 19321783 PMC2713063

[B46] Kononenko N. L. Diril M. K. Puchkov D. Kintscher M. Koo S. J. Pfuhl G.. (2013). Compromised fidelity of endocytic synaptic vesicle protein sorting in the absence of stonin 2. Proc. Natl. Acad. Sci. U S A 110, E526–E535. 10.1073/pnas.1218432110 23345427 PMC3568307

[B47] Kononenko N. L. Puchkov D. Classen G. A. Walter A. M. Pechstein A. Sawade L.. (2014). Clathrin/AP-2 mediate synaptic vesicle reformation from endosome-like vacuoles but are not essential for membrane retrieval at central synapses. Neuron 82, 981–988. 10.1016/j.neuron.2014.05.007 24908483

[B48] Koo S. J. Kochlamazashvili G. Rost B. Puchkov D. Gimber N. Lehmann M.. (2015). Vesicular synaptobrevin/VAMP2 levels guarded by AP180 control efficient neurotransmission. Neuron 88, 330–344. 10.1016/j.neuron.2015.08.034 26412491

[B49] Koo S. J. Markovic S. Puchkov D. Mahrenholz C. C. Beceren-Braun F. Maritzen T.. (2011). SNARE motif-mediated sorting of synaptobrevin by the endocytic adaptors clathrin assembly lymphoid myeloid leukemia (CALM) and AP180 at synapses. Proc. Natl. Acad. Sci. U S A 108, 13540–13545. 10.1073/pnas.1107067108 21808019 PMC3158172

[B50] Kwon S. E. Chapman E. R. (2011). Synaptophysin regulates the kinetics of synaptic vesicle endocytosis in central neurons. Neuron 70, 847–854. 10.1016/j.neuron.2011.04.001 21658579 PMC3136197

[B51] Lee J. Daniels V. Sands Z. A. Lebon F. Shi J. Biggin P. C. (2015). Exploring the interaction of SV2A with racetams using homology modelling, molecular dynamics and site-directed mutagenesis. PLoS One 10:e0116589. 10.1371/journal.pone.0116589 25692762 PMC4333566

[B52] Li Y. C. Chanaday N. L. Xu W. Kavalali E. T. (2017). Synaptotagmin-1- and synaptotagmin-7-dependent fusion mechanisms target synaptic vesicles to kinetically distinct endocytic pathways. Neuron 93, 616.e3–631.e3. 10.1016/j.neuron.2016.12.010 28111077 PMC5300960

[B53] Lynch B. A. Lambeng N. Nocka K. Kensel-Hammes P. Bajjalieh S. M. Matagne A.. (2004). The synaptic vesicle protein SV2A is the binding site for the antiepileptic drug levetiracetam. Proc. Natl. Acad. Sci. U S A 101, 9861–9866. 10.1073/pnas.0308208101 15210974 PMC470764

[B54] Ma L. Umasankar P. K. Wrobel A. G. Lymar A. McCoy A. J. Holkar S. S.. (2016). Transient Fcho1/2·Eps15/R·AP-2 Nanoclusters prime the AP-2 clathrin adaptor for cargo binding. Dev. Cell 37, 428–443. 10.1016/j.devcel.2016.05.003 27237791 PMC4921775

[B55] McMahon H. T. Bolshakov V. Y. Janz R. Hammer R. E. Siegelbaum S. A. Südhof T. C. (1996). Synaptophysin, a major synaptic vesicle protein, is not essential for neurotransmitter release. Proc. Natl. Acad. Sci. U S A 93, 4760–4764. 10.1073/pnas.93.10.4760 8643476 PMC39352

[B56] Meehan A. L. Yang X. McAdams B. D. Yuan L. Rothman S. M. (2011). A new mechanism for antiepileptic drug action: vesicular entry may mediate the effects of levetiracetam. J. Neurophysiol. 106, 1227–1239. 10.1152/jn.00279.2011 21653714 PMC3174821

[B57] Meehan A. L. Yang X. Yuan L. L. Rothman S. M. (2012). Levetiracetam has an activity-dependent effect on inhibitory transmission. Epilepsia 53, 469–476. 10.1111/j.1528-1167.2011.03392.x 22292611

[B58] Miller S. E. Sahlender D. A. Graham S. C. Honing S. Robinson M. S. Peden A. A.. (2011). The molecular basis for the endocytosis of small R-SNAREs by the clathrin adaptor CALM. Cell 147, 1118–1131. 10.1016/j.cell.2011.10.038 22118466 PMC3267021

[B59] Milosevic I. Giovedi S. Lou X. Raimondi A. Collesi C. Shen H.. (2011). Recruitment of endophilin to clathrin-coated pit necks is required for efficient vesicle uncoating after fission. Neuron 72, 587–601. 10.1016/j.neuron.2011.08.029 22099461 PMC3258500

[B60] Newell-Litwa K. Seong E. Burmeister M. Faundez V. (2007). Neuronal and non-neuronal functions of the AP-3 sorting machinery. J. Cell Sci. 120, 531–541. 10.1242/jcs.03365 17287392

[B61] Nicholson-Fish J. C. Kokotos A. C. Gillingwater T. H. Smillie K. J. Cousin M. A. (2015). VAMP4 is an essential cargo molecule for activity-dependent bulk endocytosis. Neuron 88, 973–984. 10.1016/j.neuron.2015.10.043 26607000 PMC4678114

[B62] Okamoto Y. Lipstein N. Hua Y. Lin K. H. Brose N. Sakaba T.. (2016). Distinct modes of endocytotic presynaptic membrane and protein uptake at the calyx of Held terminal of rats and mice. Elife 5:e14643. 10.7554/elife.14643 27154627 PMC4927297

[B63] Paczkowski J. E. Richardson B. C. Fromme J. C. (2015). Cargo adaptors: structures illuminate mechanisms regulating vesicle biogenesis. Trends Cell Biol. 25, 408–416. 10.1016/j.tcb.2015.02.005 25795254 PMC4475447

[B64] Pan P. Y. Marrs J. Ryan T. A. (2015). Vesicular glutamate transporter 1 orchestrates recruitment of other synaptic vesicle cargo proteins during synaptic vesicle recycling. J. Biol. Chem. 290, 22593–22601. 10.1074/jbc.m115.651711 26224632 PMC4566233

[B65] Peden A. A. Park G. Y. Scheller R. H. (2001). The Di-leucine motif of vesicle-associated membrane protein 4 is required for its localization and AP-1 binding. J. Biol. Chem. 276, 49183–49187. 10.1074/jbc.m106646200 11598115

[B66] Peng L. Tepp W. H. Johnson E. A. Dong M. (2011). Botulinum neurotoxin D uses synaptic vesicle protein SV2 and gangliosides as receptors. PLoS Pathog. 7:e1002008. 10.1371/journal.ppat.1002008 21483489 PMC3068998

[B67] Pyle R. A. Schivell A. E. Hidaka H. Bajjalieh S. M. (2000). Phosphorylation of synaptic vesicle protein 2 modulates binding to synaptotagmin. J. Biol. Chem. 275, 17195–17200. 10.1074/jbc.m000674200 10747945

[B68] Raingo J. Khvotchev M. Liu P. Darios F. Li Y. C. Ramirez D. M.. (2012). VAMP4 directs synaptic vesicles to a pool that selectively maintains asynchronous neurotransmission. Nat. Neurosci. 15, 738–745. 10.1038/nn.3067 22406549 PMC3337975

[B69] Rajappa R. Gauthier-Kemper A. Böning D. Hüve J. Klingauf J. (2016). Synaptophysin 1 clears synaptobrevin 2 from the presynaptic active zone to prevent short-term depression. Cell Rep. 14, 1369–1381. 10.1016/j.celrep.2016.01.031 26854222

[B70] Ramirez D. M. Khvotchev M. Trauterman B. Kavalali E. T. (2012). Vti1a identifies a vesicle pool that preferentially recycles at rest and maintains spontaneous neurotransmission. Neuron 73, 121–134. 10.1016/j.neuron.2011.10.034 22243751 PMC3259527

[B71] Rao Y. Rückert C. Saenger W. Haucke V. (2012). The early steps of endocytosis: from cargo selection to membrane deformation. Eur. J. Cell Biol. 91, 226–233. 10.1016/j.ejcb.2011.02.004 21458101

[B72] Robinson M. S. (2004). Adaptable adaptors for coated vesicles. Trends Cell Biol. 14, 167–174. 10.1016/j.tcb.2004.02.002 15066634

[B73] Rosenmund C. Stevens C. F. (1996). Definition of the readily releasable pool of vesicles at hippocampal synapses. Neuron 16, 1197–1207. 10.1016/s0896-6273(00)80146-4 8663996

[B74] Rost B. R. Schneider F. Grauel M. K. Wozny C. Bentz C. G. Blessing A.. (2015). Optogenetic acidification of synaptic vesicles and lysosomes. Nat. Neurosci. 18, 1845–1852. 10.1038/nn.4161 26551543 PMC4869830

[B75] Rummel A. Häfner K. Mahrhold S. Darashchonak N. Holt M. Jahn R.. (2009). Botulinum neurotoxins C, E and F bind gangliosides via a conserved binding site prior to stimulation-dependent uptake with botulinum neurotoxin F utilising the three isoforms of SV2 as second receptor. J. Neurochem. 110, 1942–1954. 10.1111/j.1471-4159.2009.06298.x 19650874

[B76] Santos M. S. Park C. K. Foss S. M. Li H. Voglmaier S. M. (2013). Sorting of the vesicular GABA transporter to functional vesicle pools by an atypical dileucine-like motif. J. Neurosci. 33, 10634–10646. 10.1523/jneurosci.0329-13.2013 23804087 PMC3693053

[B77] Schivell A. E. Batchelor R. H. Bajjalieh S. M. (1996). Isoform-specific, calcium-regulated interaction of the synaptic vesicle proteins SV2 and synaptotagmin. J. Biol. Chem. 271, 27770–27775. 10.1074/jbc.271.44.27770 8910372

[B78] Soykan T. Kaempf N. Sakaba T. Vollweiter D. Goerdeler F. Puchkov D.. (2017). Synaptic vesicle endocytosis occurs on multiple timescales and is mediated by formin-dependent actin assembly. Neuron 93, 854–866.e4. 10.1016/j.neuron.2017.02.011 28231467

[B79] Takamori S. Holt M. Stenius K. Lemke E. A. Grønborg M. Riedel D.. (2006). Molecular anatomy of a trafficking organelle. Cell 127, 831–846. 10.1016/j.cell.2006.10.030 17110340

[B80] Voglmaier S. M. Kam K. Yang H. Fortin D. L. Hua Z. Nicoll R. A.. (2006). Distinct endocytic pathways control the rate and extent of synaptic vesicle protein recycling. Neuron 51, 71–84. 10.1016/j.neuron.2006.05.027 16815333

[B81] Walther K. Diril M. K. Jung N. Haucke V. (2004). Functional dissection of the interactions of stonin 2 with the adaptor complex AP-2 and synaptotagmin. Proc. Natl. Acad. Sci. U S A 101, 964–969. 10.1073/pnas.0307862100 14726597 PMC327125

[B82] Watanabe S. Boucrot E. (2017). Fast and ultrafast endocytosis. Curr. Opin. Cell Biol. 47, 64–71. 10.1016/j.ceb.2017.02.013 28391090

[B83] Watanabe S. Liu Q. Davis M. W. Hollopeter G. Thomas N. Jorgensen N. B.. (2013a). Ultrafast endocytosis at *Caenorhabditis elegans* neuromuscular junctions. Elife 2:e00723. 10.7554/eLife.00723 24015355 PMC3762212

[B84] Watanabe S. Rost B. R. Camacho-Pérez M. Davis M. W. Söhl-Kielczynski B. Rosenmund C.. (2013b). Ultrafast endocytosis at mouse hippocampal synapses. Nature 504, 242–247. 10.1038/nature12809 24305055 PMC3957339

[B85] Watanabe S. Trimbuch T. Camacho-Pérez M. Rost B. R. Brokowski B. Sohl-Kielczynski B.. (2014). Clathrin regenerates synaptic vesicles from endosomes. Nature 515, 228–233. 10.1038/nature13846 25296249 PMC4291189

[B86] Weber T. Zemelman B. V. McNew J. A. Westermann B. Gmachl M. Parlati F.. (1998). SNAREpins: minimal machinery for membrane fusion. Cell 92, 759–772. 10.1016/s0092-8674(00)81404-x 9529252

[B87] Wen P. J. Grenklo S. Arpino G. Tan X. Liao H. S. Heureaux J.. (2016). Actin dynamics provides membrane tension to merge fusing vesicles into the plasma membrane. Nat. Commun. 7:12604. 10.1038/ncomms12604 27576662 PMC5013665

[B88] Wienisch M. Klingauf J. (2006). Vesicular proteins exocytosed and subsequently retrieved by compensatory endocytosis are nonidentical. Nat. Neurosci. 9, 1019–1027. 10.1038/nn1739 16845386

[B89] Wilhelm B. G. Mandad S. Truckenbrodt S. Kröhnert K. Schäfer C. Rammner B.. (2014). Composition of isolated synaptic boutons reveals the amounts of vesicle trafficking proteins. Science 344, 1023–1028. 10.1126/science.1252884 24876496

[B90] Willox A. K. Royle S. J. (2012). Stonin 2 is a major adaptor protein for clathrin-mediated synaptic vesicle retrieval. Curr. Biol. 22, 1435–1439. 10.1016/j.cub.2012.05.048 22727701 PMC3414847

[B91] Wu W. Wu L. G. (2007). Rapid bulk endocytosis and its kinetics of fission pore closure at a central synapse. Proc. Natl. Acad. Sci. U S A 104, 10234–10239. 10.1073/pnas.0611512104 17551019 PMC1891249

[B92] Wu W. Xu J. Wu X. S. Wu L. G. (2005). Activity-dependent acceleration of endocytosis at a central synapse. J. Neurosci. 25, 11676–11683. 10.1523/jneurosci.2972-05.2005 16354926 PMC1378116

[B93] Wu X. S. Zhang Z. Zhao W. D. Wang D. Luo F. Wu L. G. (2014). Calcineurin is universally involved in vesicle endocytosis at neuronal and nonneuronal secretory cells. Cell Rep. 7, 982–988. 10.1016/j.celrep.2014.04.020 24835995 PMC4070379

[B94] Yao J. Kwon S. E. Gaffaney J. D. Dunning F. M. Chapman E. R. (2012). Uncoupling the roles of synaptotagmin I during endo- and exocytosis of synaptic vesicles. Nat. Neurosci. 15, 243–249. 10.1038/nn.3013 22197832 PMC3435110

[B95] Yao J. Nowack A. Kensel-Hammes P. Gardner R. G. Bajjalieh S. M. (2010). Cotrafficking of SV2 and synaptotagmin at the synapse. J. Neurosci. 30, 5569–5578. 10.1523/jneurosci.4781-09.2010 20410110 PMC2866018

[B96] Yeh F. L. Dong M. Yao J. Tepp W. H. Lin G. Johnson E. A.. (2010). SV2 mediates entry of tetanus neurotoxin into central neurons. PLoS Pathog. 6:e1001207. 10.1371/journal.ppat.1001207 21124874 PMC2991259

[B97] Zhang J. Z. Davletov B. A. Südhof T. C. Anderson R. G. (1994). Synaptotagmin I is a high affinity receptor for clathrin AP-2: implications for membrane recycling. Cell 78, 751–760. 10.1016/s0092-8674(94)90442-1 8087843

[B98] Zhang N. Gordon S. L. Fritsch M. J. Esoof N. Campbell D. G. Gourlay R.. (2015). Phosphorylation of synaptic vesicle protein 2A at Thr84 by casein kinase 1 family kinases controls the specific retrieval of synaptotagmin-1. J. Neurosci. 35, 2492–2507. 10.1523/jneurosci.4248-14.2015 25673844 PMC4323530

